# Delayed Treatment with a Small Pigment Epithelium Derived Factor (PEDF) Peptide Prevents the Progression of Diabetic Renal Injury

**DOI:** 10.1371/journal.pone.0133777

**Published:** 2015-07-24

**Authors:** Alaa S. Awad, Hanning You, Ting Gao, Anzor Gvritishvili, Timothy K. Cooper, Joyce Tombran-Tink

**Affiliations:** 1 Department of Medicine, Division of Nephrology, Penn State University College of Medicine, Hershey, Pennsylvania, United States of America; 2 Department of Neural and Behavioral Sciences, Penn State University College of Medicine, Hershey, Pennsylvania, United States of America; 3 Department of Comparative Medicine, Penn State University College of Medicine, Hershey, Pennsylvania, United States of America; 4 Department of Ophthalmology, Penn State University College of Medicine, Hershey, Pennsylvania, United States of America; UCL Institute of Child Health, UNITED KINGDOM

## Abstract

Our recent publication showed that a small bioactive pigment epithelium derived factor (PEDF) peptide (P78-PEDF) prevents the development of diabetic nephropathy (DN). However, its effects on the progression of established DN were not clear. Therefore, the purpose of this study was to determine the effect of P78-PEDF in the progression of DN and to compare the effects of P78-PEDF and an ACE inhibitor (ACEi), a standard of care in DN. Experiments were conducted in *Ins2^Akita^* mice treated with P78-PEDF or captopril starting at 6 wks of age for 12 wks (early treatment) or starting at 12 wks of age for 6 wks (late treatment). We first established the optimal dose of the P78-PEDF peptide to ameliorate DN in *Ins2^Akita^* mouse for a 6 wk study period and found that the peptide was effective at 0.1- 0.5 µg/g/day. We next showed that early or late treatment with P78-PEDF resulted in protection from DN as indicated by reduced albuminuria, kidney macrophage recruitment, histological changes, inflammatory cytokines and fibrotic markers (kidney TNF-α, fibronectin, VEGFA and EGFR), and restored nephrin expression compared with vehicle-treated *Ins2^Akita^* mice. Interestingly, only early but not late treatment with captopril was as effective as P78-PEDF in reducing most DN complications, despite its lack of effect on nephrin, VEGFA and EGFR expression. These findings highlight the importance of P78-PEDF peptide as a potential therapeutic modality in both the development and progression of diabetic renal injury.

## Introduction

Diabetes mellitus (DM) is a global health problem with marked morbidity and mortality and is often complicated by micro- and macrovascular involvement, which contributes to damage to one or more target organs. Diabetic nephropathy (DN) is the most severe complication of DM worldwide and is the leading cause of end stage kidney disease, responsible for over 43% of all cases in the US and this number is likely to increase unabated [1]. Current therapies including blood pressure and glucose control and other life style changes have been only modestly successful in delaying the progression of renal failure [2]. Angiotensin converting enzyme (ACE) inhibitors (ACEi) are recognized as the standard of care in DN. However, this therapy does not result in full reversal or even fully prevent the deterioration of renal function [3]. Thus, it is important to identify new pharmacotherapeutics that will prevent or slow down the development and progression of diabetic kidney disease.

Inflammation, angiogenesis, and oxidative injury are central pathophysiological mechanisms that contribute to diabetes and DN. Pigment epithelium derived factor (PEDF) is a multifunctional, pleiotropic, secretory protein with anti-angiogenic, anti-oxidative and anti-inflammatory properties [4, 5]. PEDF plays an important role in early renal postnatal development [6]. PEDF knockout mice are both obese and diabetic [7]. PEDF acts via multiple high affinity ligands and cell receptors, although the mechanisms are not clear. There is convincing evidence that PEDF’s role in obesity and diabetes is mediated, in part, through binding to adipose triglyceride lipase (ATGL), a receptor that is essential to maintaining lipid and glucose homeostasis [8, 9]. Clinical studies have demonstrated the importance and correlation between serum PEDF levels with metabolic syndrome [10], insulin resistance [11], and renal failure [12]. In addition, a therapeutic role for PEDF in diabetic retinopathy has been clearly established [5, 13, 14]. In a recent cross sectional study, urinary PEDF was significantly increased in diabetic patients [15]. Since PEDF is a 50 kDa protein, its utility as a therapeutic agent may be limited. Recent evidence suggests that fragments of PEDF are bioactive. In particular, a 44 amino acid (AA 78–121; P78-PEDF) peptide shows excellent bioactivity in several reports [16, 17]. We have recently shown that P78-PEDF prevents the development of diabetic renal injury (primary prevention) [18], but whether P78-PEDF may also reduce the progression of DN (secondary prevention) is not clear and was the focus of this study, along with determining the optimal dose required for these activities. In addition, we compared P78-PEDF treatment to captopril (an ACEi), the standard of care for both primary and secondary prevention of DN.

Our data showed that P78-PEDF peptide treatment not only prevented the development but also progression of DN in the *Ins2* ^*Akita*^ mouse model of diabetes as indicated by reduced albuminuria, decreased kidney macrophage recruitment and inflammatory cytokines (kidney TNF-α, fibronectin, VEGFA and epidermal growth factor receptor (EGFR)), reduced histological changes, and increased nephrin expression compared to vehicle-treated *Ins2* ^*Akita*^ mice. In addition, P78-PEDF peptide but not captopril restored nephrin and reduced kidney VEGFA and EGFR expressions. These results provide evidence for use of P78-PEDF as a novel therapeutic intervention in the development and progression of diabetic renal injury.

## Materials and Methods

### Diabetic mouse models

This study was carried out in strict accordance with the recommendations in the Guide for the Care and Use of Laboratory Animals of the National Institutes of Health. The study was approved by the Penn State University College of Medicine Institutional Animal Care and Use Committee. Experiments were conducted in male D2.B6-*Ins2* ^*Akita*^/MatbJ and their wild type (WT) littermate mice (DBA/2J background; The Jackson Laboratory, stock number 007562) starting at 6 wks of age (~3 wks of diabetes). *Ins2* ^*Akita*^ mice, recommended by the Animal Models of Diabetes Complications Consortium (AMDCC) as a model of DN [19, 20], develop hyperglycemia at 3 wks of age. Mice with blood glucose levels > 350 mg/dl were considered diabetic. Mice were provided ad lib access to food and water. Urine collections were obtained by housing mice in metabolic cages. At the end of experiments, mice were euthanized by intraperitoneal injection of 100mg/kg ketamine (Henry Schein Animal Health, Dublin, OH) and 10mg/kg xylazine (Lloyd Laboratories Inc, Shenandoah, Iowa), kidneys were removed and plasma was collected for further studies.

### Drug delivery

We first established a dose response for P78-PEDF (0.01, 0.05, 0.1, 0.5 µg/g/day), a small PEDF peptide [16–18, 21] or vehicle (PBS) by continuous subcutaneous infusion beginning at 6 wks of age until 12 wks of age. We next used P78-PEDF at a dose of 0.3 μg/g/day, captopril (24 mg/L daily in drinking water; Sigma), or vehicle (PBS) via osmotic minipump starting at 6 wks (early treatment) or 12 wks (late treatment) of age until 18 wks of age.

### Blood pressure measurement

Systolic blood pressure was measured using the Coda blood pressure system (Kent Scientific Corp, Torrington, Connecticut) as previously described [18, 22–24]. Mice were habituated to the blood pressure measurement for 5 days before the experiment and then were allowed to rest quietly for 15 minutes at room temperature. All measurements were performed at the same time for each group to avoid any diurnal variations.

### Histology and immunohistochemistry

Mouse kidney tissues were fixed in 4% paraformaldehyde and embedded in paraffin. Periodic acid Schiff (PAS) staining was performed on 3 μm sections. All glomeruli (between 50–100 glomeruli) in a single transverse section for each mouse were examined under a microscope at 400× total magnification and scored individually in a blinded manner and then averaged. Semiquantitative scores (0–4+) were assigned based on blinded readings. Mesangial matrix expansion or sclerosis scoring was performed as we described previously [18, 22, 25–27]. Images were captured with an Olympus BX51 microscope and DP71 digital camera using cellSens Standard 1.12 image software. Images were obtained with 100× (oil) objective with a total magnification of 1000×. Immunohistochemistry for macrophages was performed using rat anti-mouse Mac-2 antibody (clone M3/38; Cedarlane, Burlington, NC) on paraffin sections as described previously [18, 25–27].

### Quantitative reverse transcription polymerase chain reaction (qRT-PCR)

Total RNA was isolated from kidneys using Tri reagent (Molecular Research Center, Inc, Cincinnati, OH, USA) per manufacturer’s protocol. Single-strand cDNA was synthesized using iScript cDNA Synthesis Kit (Bio-Rad, Hercules, CA) for two-step qRT-PCR. Quantitative PCR was performed using Taqman gene expression assays (TNF-α: Mm00443260_g1, fibronectin: Mm01256744_m1, VEGFA: Mm01281449_m1, EGFR: Mm00433023_m1; GAPDH: Mm99999915_g1; Life Technologies, Grand Island, NY, USA) using a Bio-Rad CFX96 Real-Time System. Data were analyzed using Bio-Rad CFX Manager Software version 2.0 and relative expression quantified using the 2^(-∆∆CT)^ equation after normalization to glyceraldehyde-3-phosphate dehydrogenase (GAPDH) as described previously [18, 22, 25, 26].

### Western blot

Kidney tissues were homogenized in lysis solution (0.1% Triton X-100) supplemented with protease inhibitor cocktail tablets (Roche). BCA protein assay (Thermo Fisher Scientific) was used to determine protein concentration. Fifteen μg of kidney lysates were separated on a NuPAGE 4~12% Bis-Tris Gel (Invitrogen) and the separated proteins transferred onto a polyvinylidene difluoride membrane (Invitrogen). After blocking using 5% non-fat dry milk, membranes were incubated with primary antibody overnight at 4°C, followed by incubation with appropriate secondary antibody for 1 hour at room temperature. Membranes were probed with primary antibodies to nephrin (1:500; Fitzgerald Industries International, cat. no. 20R-NP002) or GAPDH (1:1000; Cell Signaling Technology, Inc., cat. no. 5174S). The secondary antibody used for detection of nephrin was anti-guinea pig IgG-HRP (1:2000; Fitzgerald Industries International, cat. no. 43R-1096). Membranes were developed using enhanced chemiluminescence solutions (Thermo Fisher Scientific) followed by exposure to X-ray film and densitometry performed using Image J (NIH, http://rsbweb.nih.gov/ij/index.html) as described [18, 22, 24].

### Analytical methodology

Urine albumin was measured by ELISA using an Albuwell M kit (Exocell, Philadelphia, PA) [18, 22, 25, 27, 28]; and Body composition calculated using a LF90 Minispec Time Domain Nuclear Magnetic Resonance Spectrometer (Burker Optics, Billerica, MA) [18, 25, 27, 28].

### Statistical analysis

Comparisons among groups were performed using the SPSS version 19.0 software (SPSS, Chicago, IL) program. Data are expressed as mean ± SEM. One-way ANOVA was used when more than two groups were compared, and significance of observed differences among the groups evaluated with a least significant difference post hoc test. Statistical significance was identified at *p*< 0.05.

## Results

### P78-PEDF reduces characteristics of DN in *Ins2* ^*Akita*^ mice in a dose-dependent manner

**Fig 1:**
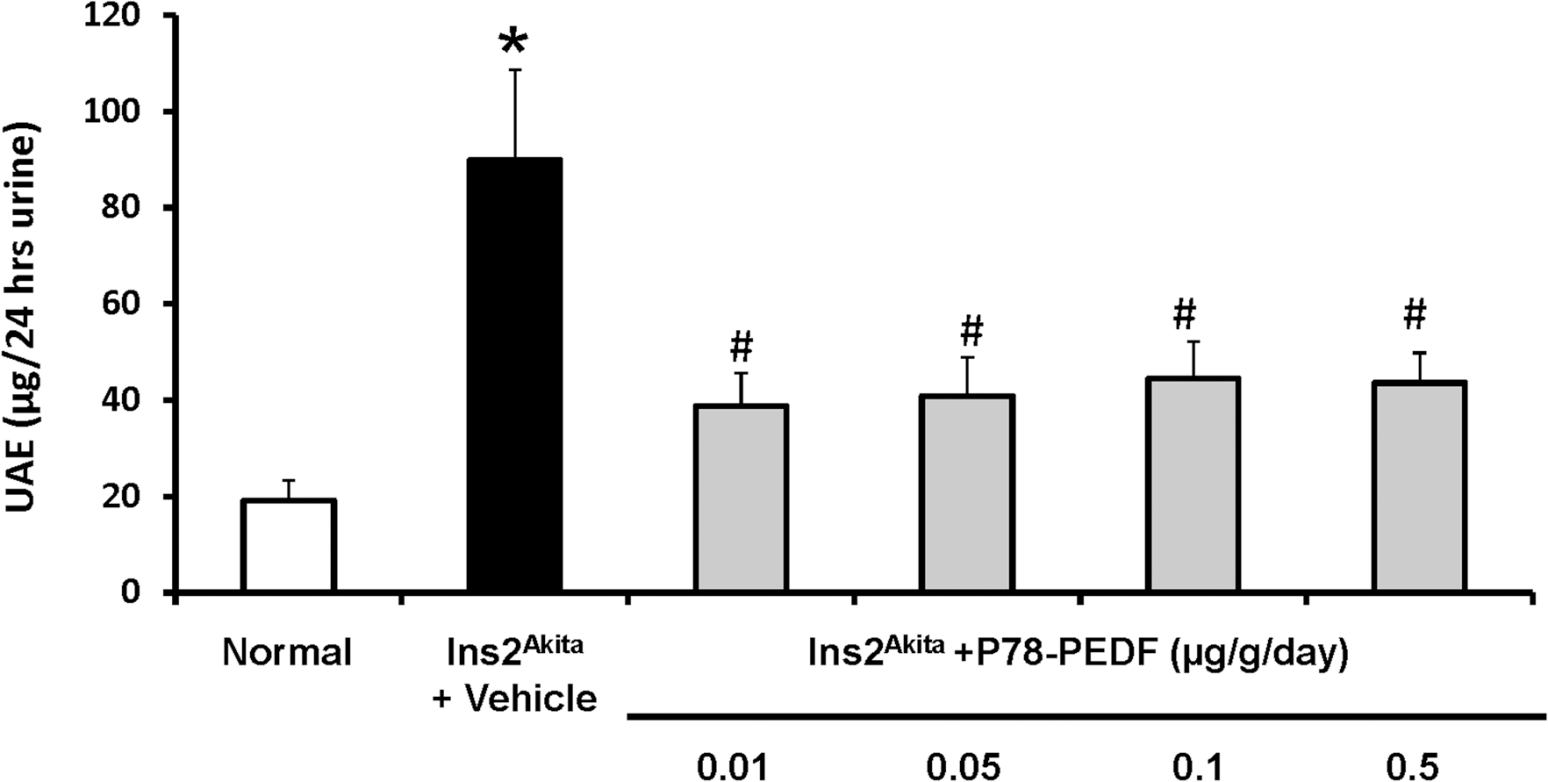
P78-PEDF peptide attenuates diabetic renal injury in *Ins2* ^*Akita*^ mice at 12 wks of age. *Ins2* ^*Akita*^ mice were treated with vehicle or P78-PEDF peptide (0.01, 0.05, 0.1, 0.5 μg/g/day) via osmotic minipump for 6 wks. Urine was collected at 12 wks of age for measurement of UAE. Results are means ± SEM. *p<0.001 compared to normal; #*p*<0.05 compared to vehicle-treated *Ins2* ^*Akita*^ mice.

We first determined the optimal dose (0.01, 0.05, 0.1, 0.5 μg/g/day) for P78-PEDF in *Ins2* ^*Akita*^ mice for 6 wks, beginning at 6 wks of age. As shown in **Table 1**, *Ins2* ^*Akita*^ vehicle-treated mice had increased blood glucose level, decreased body weight, and reduced fluid composition compared to normal mice. Treatment with P78-PEDF did not reduce blood glucose levels or blood pressure at any dose used. We also measured urine albumin excretion (UAE) as an indicator of diabetic kidney injury. Vehicle-treated *Ins2* ^*Akita*^ mice had a significant increase in UAE (**Fig 1**) compared to non-diabetic mice at 12 wks of age. Albuminuria was significantly reduced in *Ins2* ^*Akita*^ mice treated with all doses of P78-PEDF at 12 wks of age compared to vehicle treated mice.

**Table 1:** Dose-response for the effects of P78-PEDF peptide administration on diabetic *Ins2* ^*Akita*^ mice at 12 wks of age.

Treatments	Normal	*Ins2* ^*Akita*^ + Vehicle	*Ins2* ^*Akita*^ + P78-PEDF			
			0.01 μg/g/day	0.05 μg/g/day	0.1 μg/g/day	0.5 μg/g/day
**Mouse number**	10	11	10	10	13	11
**BW (g)**	28.7±1.1	25.6±0.7a	25.8±0.4a	24.1±1.2a	26.1±0.4a	26.2±0.4a
**BG (mg/dL)**	138±7	464±12c	451±17c	459±24c	451±15c	438±20c
**SBP (mmHg)**	117±3	123±4	116±3	115±3	119±2	118±3
**Fluid (%)**	7.5±0.1	5.8±0.2b	6.0±0.1b	5.8±0.2b	6.1±0.1b	6.0±0.1b

Data are mean ± SEM. a: *p*<0.05, b: *p*<0.01, c: *p*<0.0001 compared to normal. BW: body weight, BG: blood glucose, SBP: systolic blood pressure.

### P78-PEDF decreases renal histological changes in *Ins2* ^*Akita*^ mice in a dose-dependent manner

**Fig 2:**
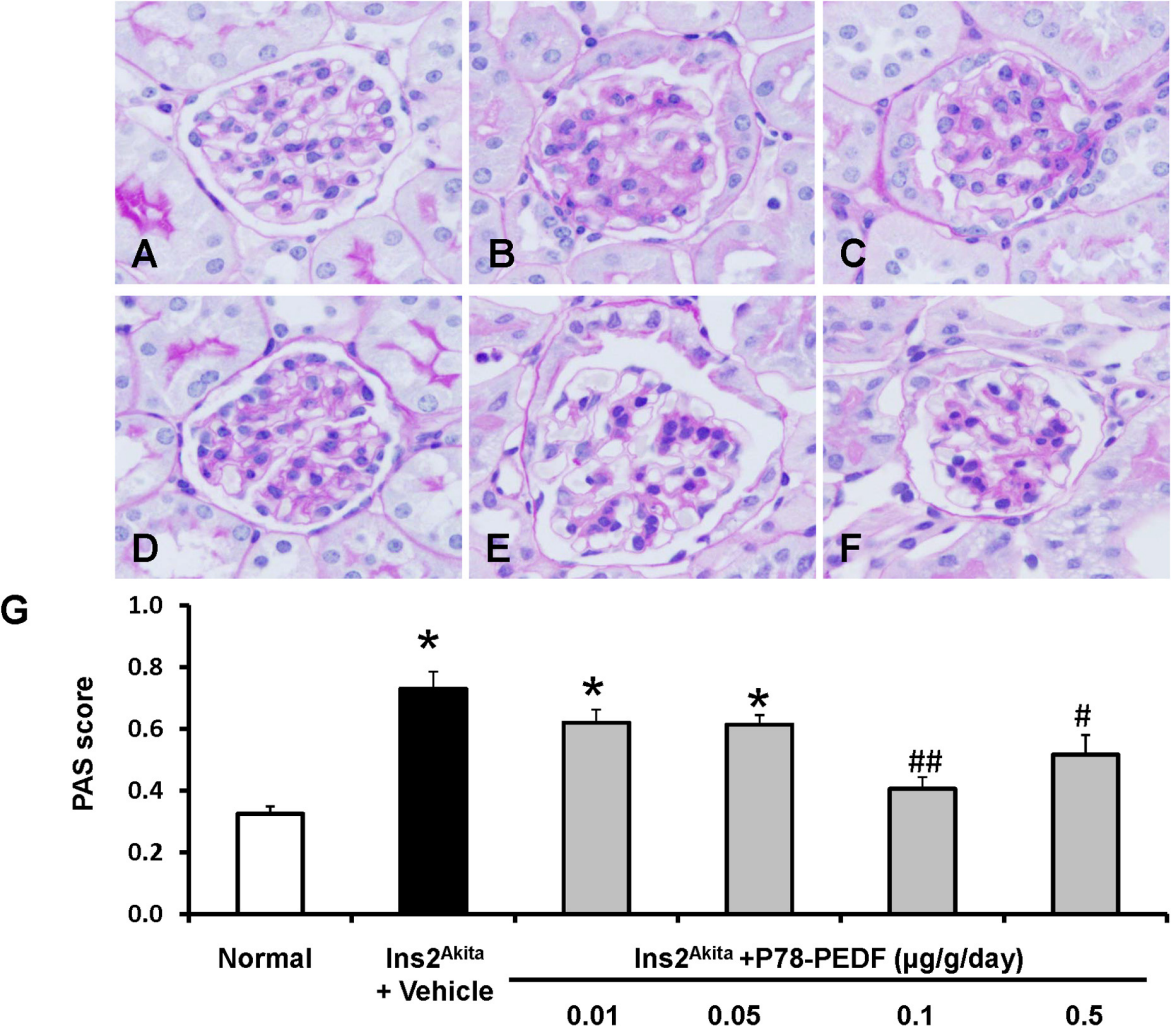
P78-PEDF peptide reduces renal histopathological changes in *Ins2* ^*Akita*^ mice at 12 wks of age. Sections were stained with PAS and all glomeruli were graded individually at 400x magnification after 12 wks of age in normal (**A**), vehicle-treated *Ins2* ^*Akita*^ (**B**) and P78-PEDF peptide-treated (Dose: μg/g/day; 0.01: **C**, 0.05: **D**, 0.1: **E**, 0.5: **F**) *Ins2* ^*Akita*^ mice. Images were taken with 100x (oil) objective with a total magnification of 1000x. Images are representative of 10–13 mice in each group. **G**: PAS score. Results are means ± SEM. *p<0.001 compared to normal; #*p*<0.05, ##*p*<0.001 compared to vehicle-treated *Ins2* ^*Akita*^ mice.

PAS staining of kidney sections **(Fig 2)** revealed increased glomerular cellularity and mesangial expansion (*p*<0.001) at 12 wks of age in vehicle-treated *Ins2* ^*Akita*^ mice vs. normal. P78-PEDF treatment in *Ins2* ^*Akita*^ mice resulted in significantly reduced glomerular cellularity and mesangial expansion at a dose of 0.1 μg/g/day (*p*<0.001) and 0.5 μg/g/day (*p*<0.05) compared to vehicle-treated *Ins2* ^*Akita*^ mice.

### P78-PEDF decreases macrophage recruitment in *Ins2* ^*Akita*^ mice in a dose-dependent manner

**Fig 3:**
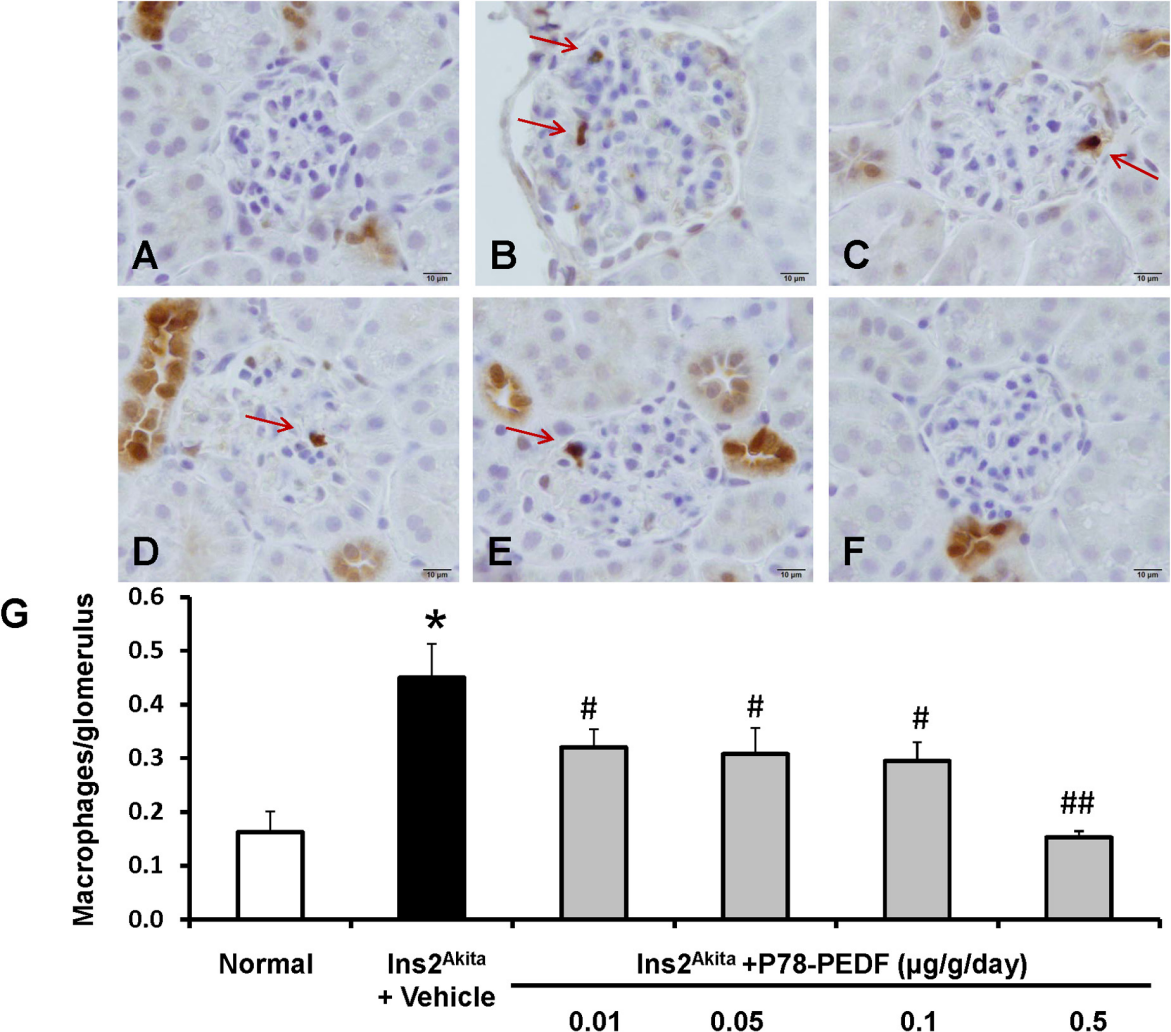
P78-PEDF peptide reduces macrophage infiltration in *Ins2* ^*Akita*^ mice at 12 wks of age. Mac-2-positive macrophages (red arrows) in glomeruli were identified by immunohistochemical staining at 12 wks of age in normal (**A**), vehicle-treated *Ins2* ^*Akita*^ (**B**) and P78-PEDF peptide-treated (Dose: μg/g/day; 0.01: **C**, 0.05: **D**, 0.1: **E**, 0.5: **F**) *Ins2* ^*Akita*^ mice. Images are representative of 40 fields from 10–13 mice in each group. **G**: The number of macrophages/glomerulus. Results are means ± SEM. *p<0.01 compared to normal; #*p*<0.05, ##*p*<0.001 compared to vehicle-treated *Ins2* ^*Akita*^ mice.

To determine whether P78-PEDF treatment is critical for kidney macrophage infiltration in DN, we examined the distribution and number of macrophages in the kidney by immunohistochemistry (Mac-2 positive macrophages) (**Fig 3**). The number of glomerular macrophages in normal mice was low but increased significantly in vehicle-treated *Ins2* ^*Akita*^ mice (*p*<0.01) at 12 wks of age. All doses of P78-PEDF treatment in *Ins2* ^*Akita*^ mice resulted in significantly reduced glomerular macrophage recruitment with an optimal dose of 0.5 μg/g/day (*p*<0.001) compared to vehicle-treated *Ins2* ^*Akita*^ mice. Given these results (**Figs 1–3)**, we used a P78-PEDF peptide dose of 0.3 μg/g/day in all subsequent experiments.

### Delayed treatment with P78-PEDF ameliorates the progression in DN of *Ins2* ^*Akita*^ mice

**Fig 4:**
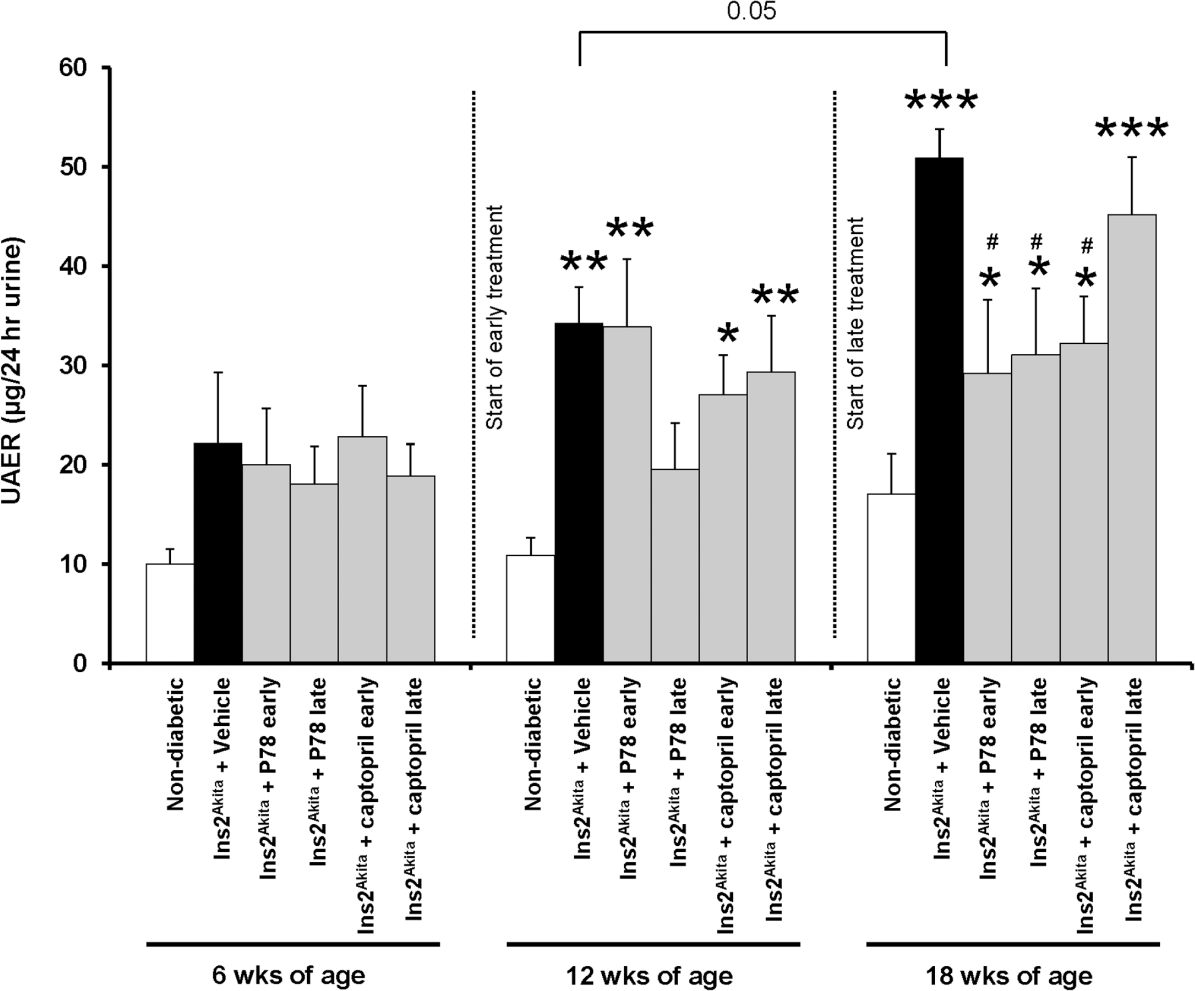
Late treatment with P78-PEDF peptide attenuates diabetic renal injury in *Ins2* ^*Akita*^ mice at 18 wks of age. *Ins2* ^*Akita*^ mice were treated with vehicle or P78-PEDF peptide (0.3 μg/g/day) via osmotic minipump or captopril (24 mg/L daily in drinking water; Sigma) starting at 6 wks (early treatment) or 12 wks (late treatment) of age. Urine was collected from each group of mice at 6, 12, and 18 wks of age for measurement of UAE. Results are means ± SEM. *p<0.05, **p<0.001, ***p<0.0001 compared to corresponding normal; #*p*<0.01 compared to corresponding vehicle-treated *Ins2* ^*Akita*^ mice.

Once we determined the optimal dose of P78-PEDF in DN, we further assessed the direct contribution of P78-PEDF in the progression of DN. *Ins2* ^*Akita*^ mice were treated with P78-PEDF (0.3 μg/g/day), captopril (24 mg/L daily in drinking water), or vehicle via osmotic minipump starting at 6 wks of age until 18 wks of age (early treatment) or at 12 wks of age until 18 wks of age (late treatment). As shown in **Table 2**, *Ins2* ^*Akita*^ vehicle-treated mice had increased blood glucose level, decreased body weight, and reduced fluid composition compared to normal mice. Early or late treatment with either P78-PEDF or captopril did not reduce blood glucose levels or blood pressure. We next measured UAE as an indicator of diabetic kidney injury. Vehicle-treated *Ins2* ^*Akita*^ mice had a significant increase in UAE (**Fig 4**) compared to normal mice at 18 wks of age. Importantly, albuminuria was significantly reduced in *Ins2* ^*Akita*^ mice with either early or late P78-PEDF treatment at 18 wks of age compared to vehicle treated mice. Interestingly, only early but not late treatment with captopril significantly reduced albuminuria to a similar extent as early treatment with P78-PEDF.

**Table 2:** Effects of early and late treatment with P78-PEDF on diabetic *Ins2* ^*Akita*^ mice at 18 wks of age.

Treatments	Normal	Ins2^Akita^ + Vehicle	Ins2^Akita^ + P78 early	Ins2^Akita^ + P78 late	Ins2^Akita^ + Captopril early	Ins2^Akita^ + Captopril late
**Mouse number**	13	8	9	7	10	10
**BW (g)**	31.1±0.8	24.3±1c	24.3±0.7c	27.0±0.4a	22.4±1.1c	24.4±0.7c
**BG (mg/dL)**						
6 wks of age	152±6	431±18c	447±16c	428±30c	477±9c	436±18c
12 wks of age	159±6	486±9c	499±1c	491±6c	483±9c	477±15c
18 wks of age	159±4	498±2c	499±1c	499±1c	494±4c	490±8c
**SBP (mmHg)**	119±3	115±2	121±3	120±4	119±4	114±5
**Fluid (%)**	7.5±0.1	6.1±0.3c	6.5±0.3a	6.4±0.3b	5.8±0.1c	6.1±0.2c

Data are mean ± SEM. a: *p*<0.01, b: *p*<0.001, c: *p*<0.0001 compared to normal. BW: body weight, BG: blood glucose, SBP: systolic blood pressure.

### Delayed treatment with P78-PEDF decreases renal histopathological changes in DN of *Ins2* ^*Akita*^ mice

**Fig 5:**
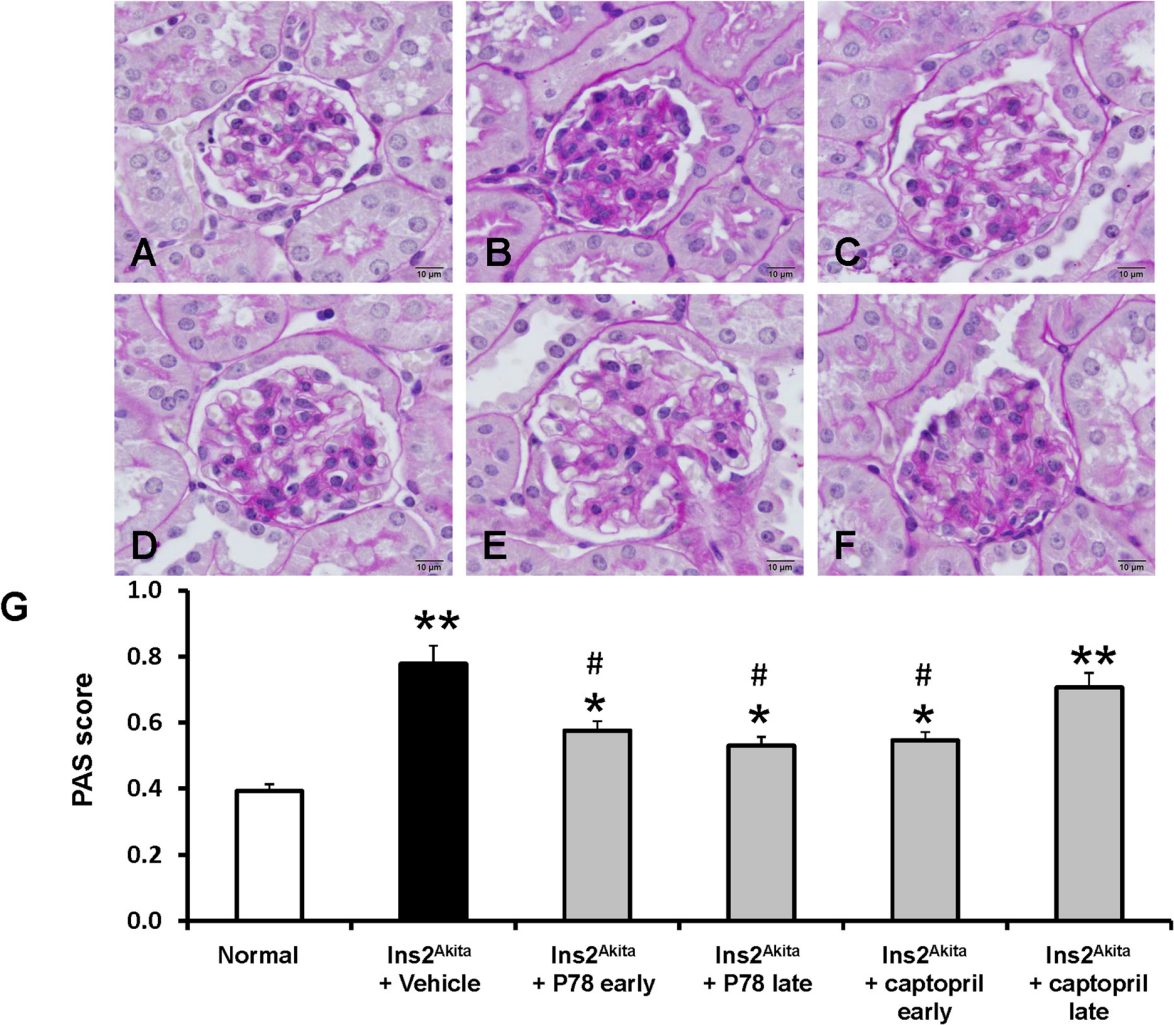
Late treatment with P78-PEDF peptide reduces renal histopathological changes in *Ins2* ^*Akita*^ mice at 18 wks of age. Sections were stained with PAS and all glomeruli were graded individually at 400x magnification after 18 wks of age in normal (**A**), vehicle-treated *Ins2* ^*Akita*^ (**B**), P78-PEDF peptide early treated **(C)**, P78-PEDF peptide late treated **(D)**, captopril early treated (**E)**, or captopril late treated (**F)** *Ins2* ^*Akita*^ mice. Images were taken with 100x (oil) objective with a total magnification of 1000x. Images are representative of 7–13 mice in each group. **G**: PAS score. Results are means ± SEM. *p<0.01, **p<0.001 compared to normal; #*p*<0.01 compared to vehicle-treated *Ins2* ^*Akita*^ mice.

PAS staining of kidney sections **(Fig 5)** revealed increased glomerular cellularity and mesangial expansion (*p*<0.001) at 18 wks of age in vehicle-treated *Ins2* ^*Akita*^ mice. Early or late treatment with P78-PEDF at 18 wks of age in *Ins2* ^*Akita*^ mice resulted in significantly reduced glomerular cellularity and mesangial expansion compared to vehicle-treated *Ins2* ^*Akita*^ mice. In contrast, only early but not late treatment with captopril was comparable to the same treatment with P78-PEDF in significantly reducing glomerular cellularity and mesangial expansion.

### Delayed treatment with P78-PEDF decreases macrophage recruitment in DN of *Ins2* ^*Akita*^ mice

**Fig 6:**
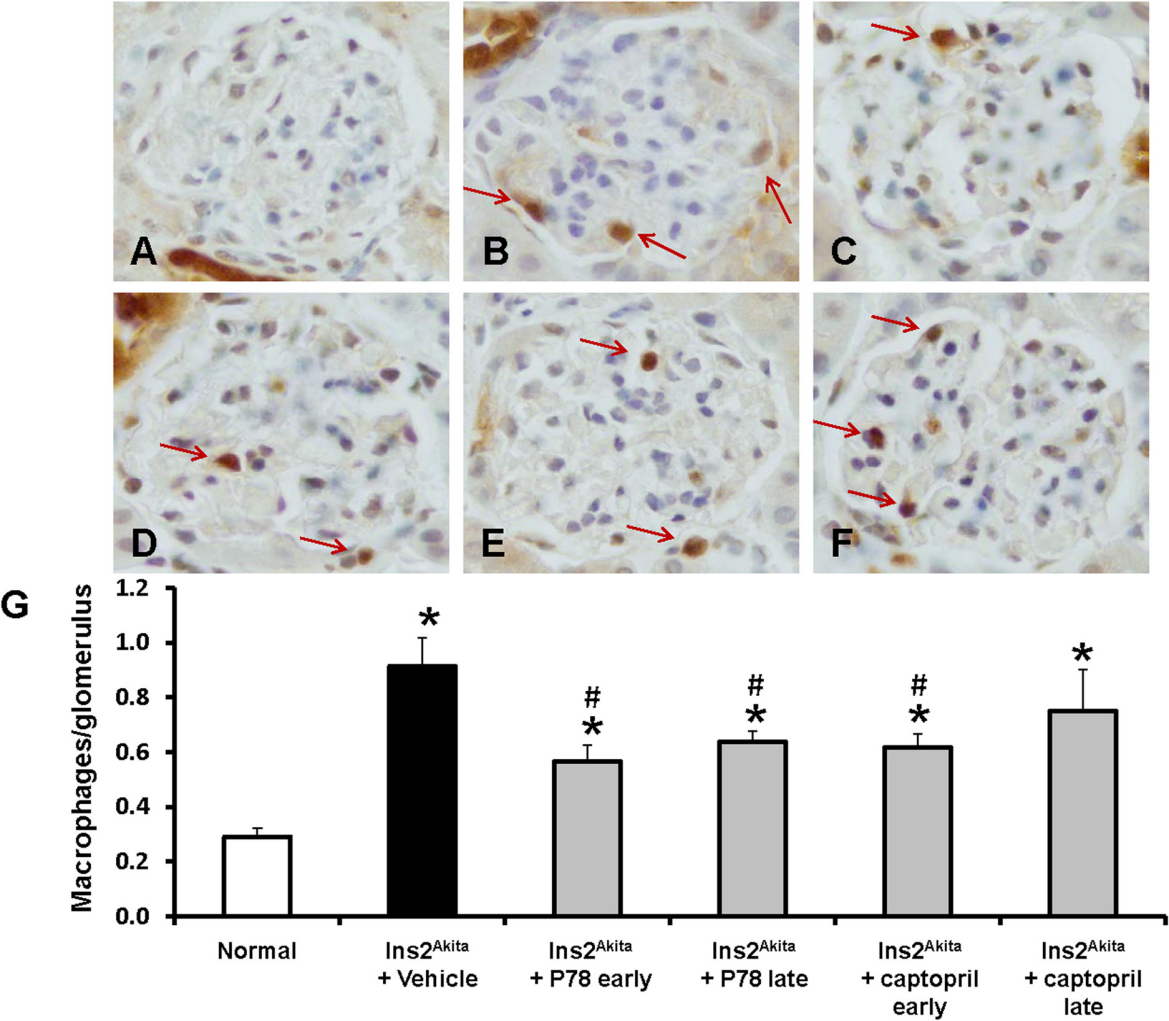
Late treatment with P78-PEDF peptide reduces macrophage infiltration in *Ins2* ^*Akita*^ mice at 18 wks of age. Mac-2-positive macrophages in glomeruli (red arrows) were identified by immunohistochemical staining at 18 wks of age in normal (**A**), vehicle-treated *Ins2* ^*Akita*^ (**B**), P78-PEDF peptide early treated **(C)**, P78-PEDF peptide late treated **(D)**, captopril early treated (**E)**, or captopril late treated (**F)** *Ins2* ^*Akita*^ mice. Images are representative of 40 fields from 7–13 mice in each group. **G**: The number of macrophages/glomerulus. Results are means ± SEM. *p<0.01 compared to normal; #*p*<0.05 compared to vehicle-treated *Ins2* ^*Akita*^ mice.

To determine the extent to which P78-PEDF treatment was effective in reducing progression of DN as it does in the development of DN by reducing kidney macrophage infiltration, we examined distribution and number of macrophages in the kidney by immunohistochemistry (Mac-2 positive macrophages) (**Fig 6**). The number of glomerular macrophages in normal mice was low and increased significantly in vehicle-treated *Ins2* ^*Akita*^ mice (*p*<0.01) at 18 wks of age. Early or late treatment with P78-PEDF at 18 wks of age in *Ins2* ^*Akita*^ mice resulted in significantly reduced glomerular macrophage recruitment compared to vehicle-treated *Ins2* ^*Akita*^ mice. In contrast, only early treatment with captopril was comparable to early treatment with P78-PEDF in significantly reducing glomerular macrophage recruitment. Late captopril treatment was less effective than late P78-PEDF treatment.

### Delayed treatment with P78-PEDF decreases inflammatory cytokines and fibrotic markers in DN of *Ins2* ^*Akita*^ mice

**Fig 7:**
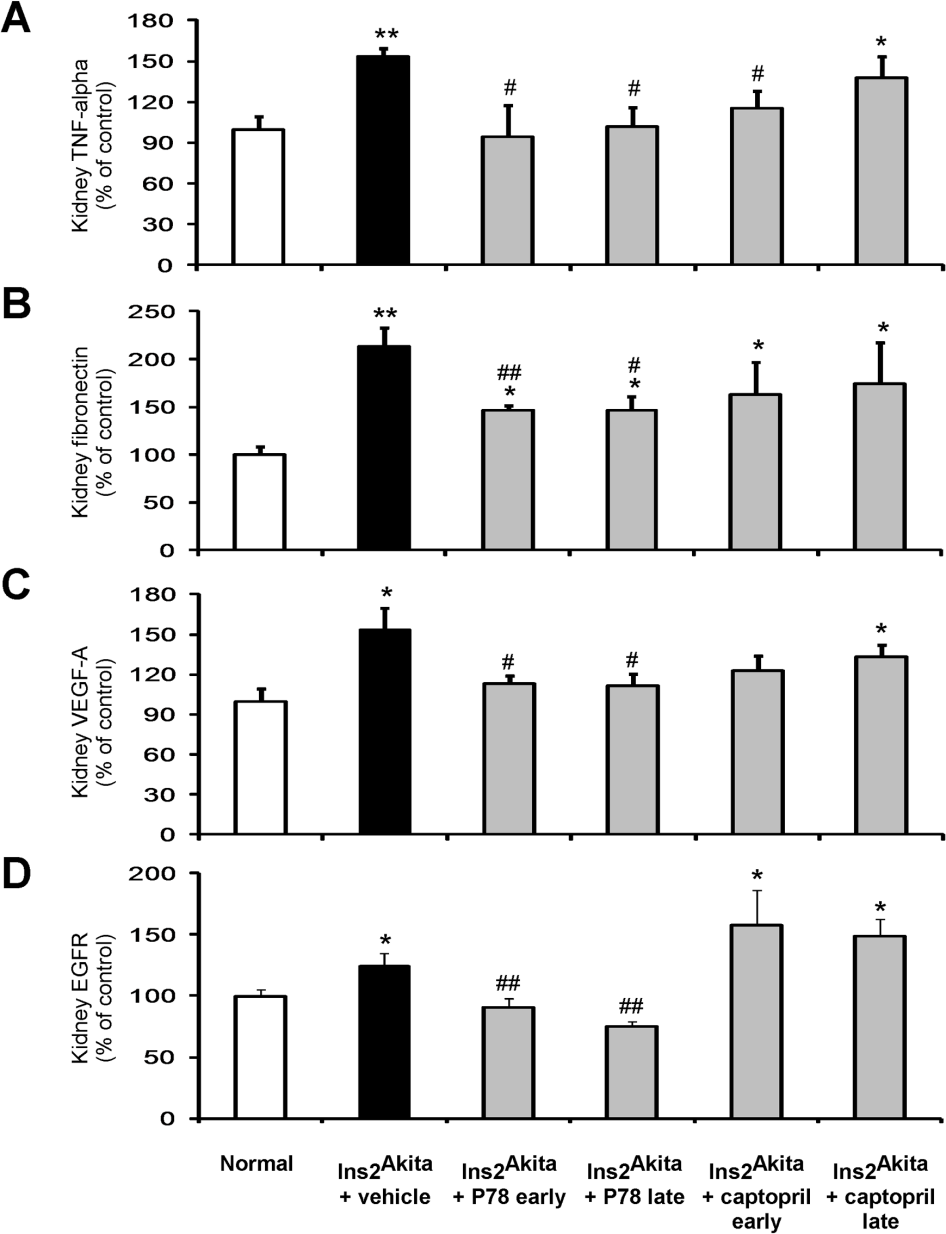
Late treatment with P78-PEDF peptide reduces inflammatory cytokines and fibrotic markers in *Ins2* ^*Akita*^ mice at 18 wks of age. RT-PCR was performed on whole mouse kidney total RNA at 18 wks of age. TNF-α **(A)**, fibronectin **(B)**, VEGFA **(C),** and EGFR **(D)** mRNA expression were normalized with GAPDH. Results are means ± SEM. *p<0.05, **p<0.01 compared to normal; #*p*<0.05, ##*p*<0.01 compared to *Ins2* ^*Akita*^+vehicle.

Increased inflammatory cytokines and fibrotic markers are major features of and important predictors of DN [18, 29, 30]. Therefore, we further assessed the anti-inflammatory and anti-fibrotic effects of P78-PEDF treatment in diabetic mice **(Fig 7)**. Kidney TNF-α **(Fig 7A)**, fibronectin **(Fig 7B)**, VEGFA **(Fig 7C),** and EGFR **(Fig 7D)** mRNA expression were significantly increased in vehicle-treated *Ins2* ^*Akita*^ mice at 18 wks of age compared to normal mice. Both early and late treatments of *Ins2* ^*Akita*^ mice at 18 wks of age with P78-PEDF significantly reduced kidney TNF-α, fibronectin, VEGFA, and EGFR mRNA expression levels compared to vehicle-treated *Ins2* ^*Akita*^ mice. In contrast, only early but not late treatment with captopril significantly reduced kidney TNF-α mRNA expression without affecting kidney fibronectin, VEGFA or EGFR mRNA expression levels.

### Delayed treatment with P78-PEDF prevents reduction in nephrin protein expression in *Ins2* ^*Akita*^ mice

**Fig 8:**
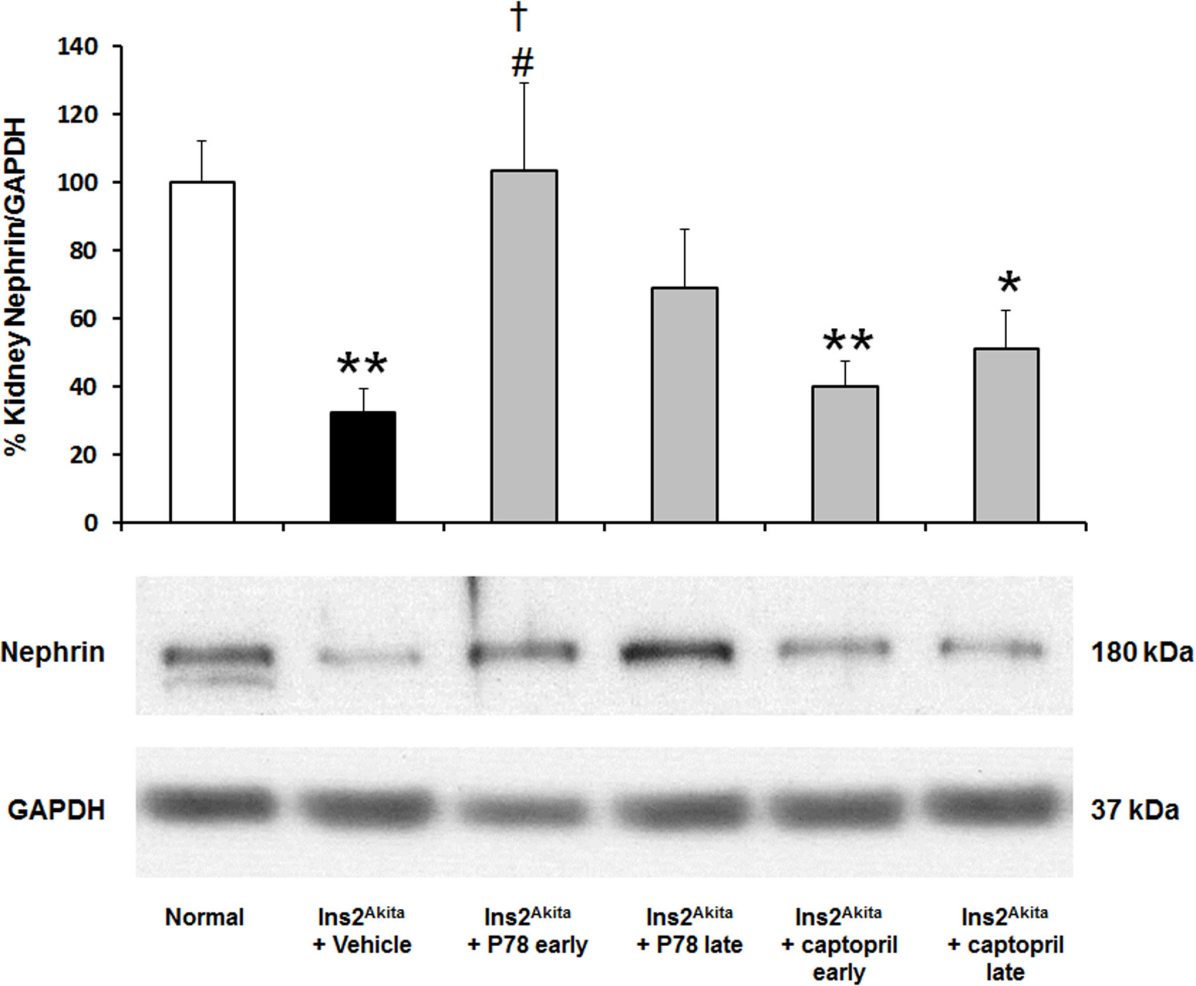
Late treatment with P78-PEDF peptide restores renal nephrin protein expression in *Ins2* ^*Akita*^ mice at 18 wks of age. Kidney nephrin expression was detected using western blot at 18 wks of age. Quantification was performed by densitometry followed by normalization to GAPDH. Results are means ± SEM. *p<0.01, **p<0.01 compared to normal; #*p*<0.05 compared to vehicle-treated *Ins2* ^*Akita*^ mice; †*p*<0.05 compared to *Ins2* ^*Akita*^ mice treated with captopril early.

Nephrin is a critical component protein in the glomerular slit diaphragm, which plays a pivotal role in maintaining kidney function. A decrease in nephrin expression is associated with albuminuria. As shown in **Fig 8**, nephrin protein expression was significantly reduced in vehicle-treated *Ins2* ^*Akita*^ mice at 18 wks of age compared to normal mice. Early treatment with P78-PEDF in *Ins2* ^*Akita*^ mice resulted in significantly increased nephrin protein expression compared to vehicle-treated *Ins2* ^*Akita*^ mice of the same age while late P78-PEDF treatment was not significantly different compared to normal mice. In contrast, neither early nor late treatment with captopril affected nephrin protein expression, which was comparable in levels to vehicle-treated *Ins2* ^*Akita*^ mice.

## Discussion

Despite major efforts to understand the pathogenesis of DN, the disease still progresses in spite of current therapeutic measures, such as control of blood pressure and blood glucose levels and use of renin-angiotensin-aldosterone system inhibitors [2]. Identification of other DN targets may lead to more effective treatments or prevention of disease progression. Accumulating evidence suggests that PEDF is one such factor in developing treatments for diabetic renal injury. PEDF has well-established anti-angiogenic, anti-oxidative and anti-inflammatory actions in the eye [4, 5], yet its role in diabetic kidney injury is not completely clear. We have shown previously that P78-PEDF treatment confers kidney protection in preventing DN [18]; however that study was only focused on early stage (development) DN. Whether P78-PEDF treatment is also important in delaying the progression of DN was not clear. Understanding the role of P78-PEDF in treating (rather than preventing) DN will have clinical relevance. The current study shows that P78-PEDF prevents the development and progression of DN in the *Ins2* ^*Akita*^ mice, a well-established model of type-1 diabetes. This conclusion is based on its ability to reduce albuminuria, histopathological changes, kidney macrophage recruitment, inflammatory cytokines, and fibrotic markers associated with diabetes. Furthermore, both early and late treatment with P78-PEDF preserved expression of the podocyte structural protein nephrin during diabetes. These findings reveal an important role for P78-PEDF peptide and/or other PEDF peptides in the development and progression of DN. Thus, P78-PEDF is a potentially new therapeutic tool for treating diabetic patients.

The PEDF protein is expressed in several tissues and cell types including the postnatal kidney [6]. In addition, we have shown that PEDF is expressed in the kidney vasculature, interstitial spaces, glomeruli, medulla and tubular epithelial cells, and glomerular endothelial cells [18] and that the protein and mRNA levels were markedly reduced in diabetic mice [18] and rat kidney [31].

In this study, the renal protective effects of early or late treatment with P78-PEDF peptide correlate with a significant reduction in kidney macrophage infiltration. Infiltrating macrophages can release lysosomal enzymes, nitric oxide, reactive oxygen species, transforming growth factor-beta, vascular endothelial growth factor and cytokines such as TNF-α, interleukin-1 and interferon (IFN)-γ [32], which could play a pivotal role in the development and progression of DN. We have shown previously that early treatment with P78-PEDF peptide significantly reduced the increase in urinary TNF-α and kidney VEGFA protein levels [18]. The current study confirms these results and shows that early or late treatment with P78-PEDF significantly reduced kidney TNF-α, and VEGFA expressions. TNF-α is produced mainly by monocytes/macrophages and is associated with increasing vascular endothelial permeability in diabetes mellitus [33]. PEDF has been shown to counteract the effects of VEGF [34]. Increased VEGF can lead to glomerular hypertrophy and proteinuria [35], and thickening of glomerular basement membrane [36, 37]. Both early and late treatment with the P78-PEDF peptide also resulted in less mesangial expansion and glomerular hypercellularity in diabetes and was associated with reduced fibronectin mRNA expression, indicating a possible association between PEDF reduction in the diabetic kidney with initiation and/or progression of diabetic renal fibrosis.

Since ACEi is the gold standard for treating diabetic patients, every potential new therapy should be compared to it as standard of care. Our data show that early treatment with captopril, an ACEi, was almost as effective as early treatment with P78-PEDF in reducing albuminuria, kidney macrophages recruitment, and histopathological changes along with reduced kidney TNF-α expression. Early treatment with captopril however, lacks any effects on kidney VEGFA, fibronectin or nephrin expression levels. Interestingly, late treatment with captopril lacks any effect to ameliorate diabetic renal injury. Additional studies to investigate the role of combined P78-PEDF and captopril treatments will be needed in future study.

The mechanism by which P78-PEDF peptide mediates protection in DN is not completely known. We have shown previously, that P78-PEDF peptide may have a direct effect on podocytes [18]. Podocytes play a key role in the maintenance of the glomerular filtration barrier [38], and normal podocyte function is intimately linked to its complex cytoskeletal architecture. The effects of early or late treatment with P78-PEDF to restore nephrin protein expression further support this conclusion. In addition, our data show increased EGFR expression in vehicle-treated *Ins2* ^*Akita*^ mice at 18 wks of age. Both early and late treatments of *Ins2* ^*Akita*^ mice at 18 wks of age with P78-PEDF significantly reduced kidney EGFR expression levels compared to vehicle-treated *Ins2* ^*Akita*^ mice. EGFR is widely expressed and induced in the kidney [39, 40]. Inhibition of EGFR has been shown to prevent DN [41]. We also speculate that macrophage infiltration plays a major role in PEDF-mediated renal tissue protection in DN. This conclusion is based on our results using diabetic CD11b-DTR mice, in which human DTR expression is under the control of the CD11b promoter [18]. Our data show that macrophage depletion in diabetic CD11b-DTR mice using diphtheria toxins restored PEDF protein expression to normal levels (**S1 Fig).** Additional studies to investigate the direct interaction between macrophages and PEDF will be needed in future.

In conclusion, our study demonstrates that treatment with P78-PEDF not only prevents the development but also the progression of DN. Results of this study may ultimately lead to novel therapeutic interventions using P78-PEDF peptide in the treatment of diabetic kidney disease.

## Supporting Information

S1 Fig Macrophage depletion in diabetic CD11b-DTR mice restores PEDF protein expression to normal levels. Male 6-wk-old B6.FVB-Tg(ITGAM-DTR/EGFP)34Lan/J (CD11b-DTR) mice (stock no. 006000, Jackson Laboratory, Bar Harbor, MN) were given multiple intraperitoneal injections of vehicle or STZ. A diphtheria toxin (DT) (List Biological Laboratories) was injected intraperitoneally at the dose of 25 ng/g body wt weekly for 6 wk after STZ injection [25]. Kidney tissues were homogenized in lysis solution as indicated in the Method section for western blot analysis of PEDF expression as we described previously [18]. Quantification was performed by densitometry followed by normalization to GAPDH. Results are means ± SEM for **n** = 4–5 mice in each group. *p<0.05 compared to diabetic CD11b-DTR mice treated with diphtheria toxins.
(PDF)
