# Supplementary figures and images for: Delayed Treatment with a Small Pigment Epithelium Derived Factor (PEDF) Peptide Prevents the Progression of Diabetic Renal Injury
Source: PLoS One. 2015 Jul 24;10(7):e0133777. doi: 10.1371/journal.pone.0133777 (PMC4514848; doi:10.1371/journal.pone.0133777)

Supplemental Figure 1

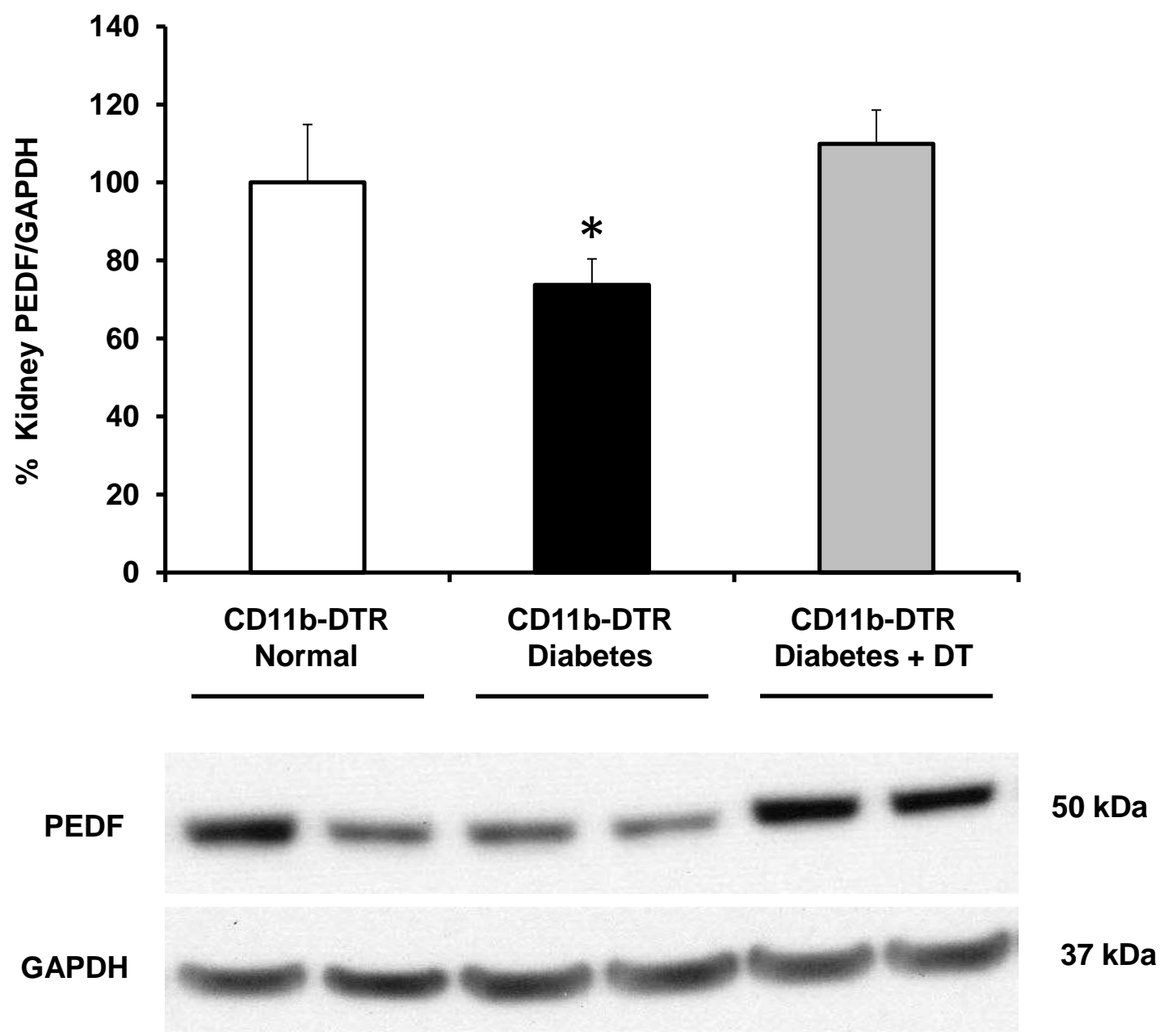

Supplement: S1 Fig — Male 6-wk-old B6.FVB-Tg(ITGAM-DTR/EGFP)34Lan/J (CD11b-DTR) mice (stock no. 006000, Jackson Laboratory, Bar Harbor, MN) were given multiple intraperitoneal injections of vehicle or STZ. A diphtheria toxin (DT) (List Biological Laboratories) was injected intraperitoneally at the dose of 25 ng/g body wt weekly for 6 wk after STZ injection [25]. Kidney tissues were homogenized in lysis solution as indicated in the Method section for western blot analysis of PEDF expression as we described previously [18]. Quantification was performed by densitometry followed by normalization to GAPDH. Results are means ± SEM for n = 4–5 mice in each group. *p<0.05 compared to diabetic CD11b-DTR mice treated with diphtheria toxins. (PDF) [file pone.0133777.s001.pdf]
